# Effect of Different Processing Methods on the Accumulation of the Phenolic Compounds and Antioxidant Profile of Broomcorn Millet (*Panicum miliaceum* L.) Flour

**DOI:** 10.3390/foods8070230

**Published:** 2019-06-27

**Authors:** Md Obyedul Kalam Azad, Da In Jeong, Md Adnan, Timnoy Salitxay, Jeong Won Heo, Most Tahera Naznin, Jung Dae Lim, Dong Ha Cho, Byoung Jae Park, Cheol Ho Park

**Affiliations:** 1 Department of Bio-Health Technology, College of Biomedical Science, Kangwon National University, Chuncheon 24341, Korea; 2 Head of Research and Technology, Rentia Plant Factory, Chuncheon 24341, Korea; 3 Laos-Korea Science and Technology Center, Souphanouvong University, Luangprabang 0603, Laos; 4 Department of Biosystems and Technology, Swedish University of Agricultural Sciences, Box 103 23053 Alnarp, Sweden; 5 Department of Herbal Medicine Resource, Kangwon National University, Samcheok 25949, Korea; 6 Faculty of Agriculture, Kagoshima University, Kagoshima 890-0065, Japan

**Keywords:** broomcorn millet, processing methods, total phenolic, flavonoid, antioxidant capacity

## Abstract

Broomcorn millet (*Panicum miliaceum* L.) is an important nutritious ancient minor-cereal food crop. However, this crop is little explored in the food processing arena to improve its functionality. In this context, different processing methods were applied to enhance the secondary compounds of broomcorn millet. Four different individual methods such as roasting, steaming, puffing, and extrusion were applied at 110 °C to enhance the functional attributes of millet flour. It was observed that the significantly highest content of total phenolic (TP) (670 mg/100 g of ferulic acid equivalent) and total flavonoid (TF) (391 mg/100 g of rutin equivalent ) was attained in the roasted whole millet followed by steaming (315 mg/100 g, 282 mg/100 g), puffing (645 mg/100 g, 304 mg/100 g), extrusion (455 mg/100 g, 219 mg/100 g), and control (295 mg/100 g, 183 mg/100 g). The chromatographic analysis showed a greater content of single phenolic acids such as syringic acid, gallic acid, 4-hydroxy benzoic acid, ferulic acid, sinapic acid, and catechin in roasted millet compared to control, and the content of each acid was higher in whole millet than dehulled. Results also indicated that the content of ferulic acid was relatively higher among the quantified single phenolic acid from broomcorn millet. Likewise, in comparison with dehulled millet, the roasted whole millet showed higher total antioxidant capacity, measured by the 2,2-diphenyl-1 picryl hydrazyl (DPPH), the ferric reducing antioxidant power assay (FRAP), the phosphomolybdenum method (PPMD), and the hydroxyl radical scavenging capacity (HRSC) method. Lastly, it is concluded that the roasting method should be taken into consideration in the processing of broomcorn millet to enhance the content of nutraceutical compounds and improve its functionality.

## 1. Introduction

Broomcorn millet, known by other names such as proso millet, common millet, and hog millet (*Panicum miliaceum* L.), is a minor cereal food crop that has traditionally been cultivated in Korea since ancient times. It is an essential ecological food security crop known for its nutritional quality and can provide subsistence for nutrient-scarce people [1]. Moreover, millets serve as a major food component in many African and Asian populations, specifically among non-affluent segments in their respective societies [2].

Epidemiological studies show that increased consumption of millet is associated with age-related challenges, viz. reduced cholesterol, heart-related disease [3], diabetes [4], and liver disorder [5]. Polyphenols can improve gut health and reduce the risk of coronary heart disease and have anti-inflammatory, antimutagenic, anticarcinogenic, and antioxidant activities [6,7]. It is generally believed that antioxidants scavenge free radicals and reactive oxygen species and thus inhibit oxidative mechanisms, which lead to degenerative diseases [8]. Millets have more than fifty phenolic compounds such as phenolic acids and their derivatives, flavonols, flavones, and flavanonols [2], which possess a high antioxidant capacity [7]. It has been identified that phenolic compounds in cereal foods are present either in soluble or bound form. Soluble phenolic compounds are mostly available in the outer layers of the kernel, and bound compounds are present within the cell wall [9]. Phenolic compounds that are extractable into aqueous media are generally referred to as free and non-conjugated compounds. The leftover residue after extraction of the soluble phenolics is referred to as conjugated and bound compounds [10]. 

Zhang et al. [11] reported that the phenolic acids of broomcorn millet are generally present in the bound form. The major phenolic compounds in millet are hydroxybenzoic acid derivatives including syringic acid, gallic acids, vanillic acids, 4-hydroxyl benzoic acid, and hydroxycinnamic acid derivatives including caffeic acid, ferulic acid, and sinapic acid [12,13]. Among the phenolic acids, hydroxycinnamic acids are the most abundantly found in broomcorn millet, finger millet, and foxtail millet [14]. The hydroxycinnamic acids found in broomcorn millet include ferulic acid, chlorogenic acid, caffeic acid, and coumaric acid. However, ferulic acid is found in bound form in broomcorn millet and finger millet, while it is found in free form in pearl millet [11]. Chadrasekara and Shahidi [12] reported that ferulic acid is a major phenolic compound that plays a vital role in the spectacular antioxidant capacity of millets. Other phenolic acids in millets are gallic acid, p-hydroxy benzoic acid, vanillic acid, caffeic acid, sinapic acid, syringic acid, and many more. It has been noted that ferulic acid prevents cancers in humans [15], and caffeic acid selectively blocks the biosynthesis of leukotrienes, while syringic acid exhibits a hepatoprotective effect [16]. 

Processed food is currently getting special attention owing to its enhanced functionality and superior health benefits. The availability of nutrients and phenolic compounds are likely to be influenced by the processing methods. It has been reported that pre-treatment/mechanical treatment make phenolic compounds free from their bound form. Therefore, various processing technologies have been used to facilitate the release and to increase the accessibility of bound phenolic compounds in cereal grains [17]. Many studies reported that pretreatment such as dehulling or milling has a significant positive effect on the nutrient content of the millet flour [18,19]. Several food processing methods, such as thermal processing, sprouting, and fermentation, is being used to enhance secondary bioactive compounds’ profiles [20]. Geetha et al. [21] and Fei et al. [22] demonstrated that steaming and cooking processes enhance the phenolic content and antioxidant profile of foxtail and proso millet. In the food industry, roasting is used to improve the bioaccessibility of secondary compounds (18). Roasting causes both desirable and undesirable changes in the physical, chemical, and nutritional properties of the plant foods [19]. Roasting is used to deactivate anti-nutritional components in food materials and to provide a characteristic flavor and brown color to final products [22]. Extrusion may help break down the high molecular weight of secondary compounds into their lower molecular weight constituents that have higher bioaccessibility [23]. Several studies on cereals have reported that thermal treatments may reduce the phenolic content and their antioxidant activities, depending on the severity of heat treatment, time of exposure, and the type of cereal tested [24,25].

Although millet is known as a nutritious cereal, it is little explored for the possibility of developing novel food products using different processing technologies [21]. Much attention has been given in the investigation of sorghum, finger millet, pear millet, and foxtail millet [8,11,26]. However, information on the processing of broomcorn millet to increase functional quality is rather scarce. The neglected broomcorn millet can be explored for value addition by the application of the processing method. To elucidate the significance of the polyphenols from millet, it is necessary to explore the amount of polyphenols present in the food materials and consumed in the diet. In this regard, different processing methods such as roasting, puffing, steaming, and extrusion were applied to enhance the phenolic compounds and antioxidant capacity of broomcorn millet flour.

## 2. Materials and Methods

### 2.1. Preparation of Broomcorn Millet

Broomcorn millet was purchased from Samcheok County, Korea. The millet was dehulled two times by the huller under the power of 1.8 kW. After the dehulling, the ratio of hull removal was more than 98%. The millet sample used in this study was free from disease and insect infections, well representative that there was no contamination.

### 2.2. Sample Preparation

*Roasting:* About 300 g of millet seeds (whole and dehulled) were soaked in water (5% *w/v*) for six h in the dark and then roasted in a toaster (Electrolux, Guangzhou, China) for 10 min at 110 °C. The roasted seeds were cooled and then milled with a coffee mill. The flour was passed through a 200-µm sieve and stored at 4 °C for further analysis.

*Steaming:* About 300 g of soaked millet whole and dehulled seeds (millet: water = 1:5 *w/w*) were put in an automatic steamer pot (Hamilton Beach Digital Food Steamer) for 10 min at 110 °C. Steamed millet was dried in an oven at 50 °C and milled with a coffee mill. The flour was passed through a 200-µm sieve and stored at 4 °C for further analysis.

*Puffing:* Puffing was done by using a puffing machine at 110 °C and 1.0MPa. The puffed seeds were then milled with a coffee mill. The flour was passed through a 200-µm sieve and stored at 4 °C for further analysis.

*Extrusion:* The millet extrudate was prepared using an STS-25HS twin-screw hot melt extruder (HME) (Hankook E.M. Ltd., Pyeongtaek, Korea). The extruder was equipped with a round-shaped die (1 mm) and was operated at a feeding rate of 30 g/min, 150 rpm with high shear. The temperature profile from the feeding zone to die was 110 °C with a pressure of 80–100 bar. Once a steady temperature was attained in the extruder zone (10 min after switching on the HME), 300 g of millet flour added with 20% moisture content were fed through the feeder. The millet extrudate was dried in an oven at 50 °C, then milled with a coffee mill. The flour was passed through a 200-µm sieve to obtain a uniform particle size. The millet flour was put in zip-lock covers and stored at 4 °C for further analysis.

### 2.3. Extraction of Millet Flour

All the treated millet flours were freeze-dried for 24 h to achieve uniform moisture content <4% among the samples before extraction. In brief, millet flour (1 g) was dissolved in 100 mL of ethanol (80%, *v*/*v* in water) and then sonicated at 35 °C for 60 min. Afterward, the sonicated extracts were filtered (Advantech 5B filter paper, Tokyo Roshi Kaisha Ltd., Saitama, Japan), followed by evaporation (Rotatory Evaporator, EYLA N-1000, Tokyo, Japan) at 40 °C to get yields (crude extracts) that were freeze-dried to maintain moisture content <3%. Finally, the stock solution (1 mg/L) was prepared by diluting the dried powder using distilled water and kept in a refrigerator (−20 °C) for further investigation.

### 2.4. Estimation of Total Phenolic Content

Folin–Ciocalteu method was performed to estimate the phenolic content of the processed millet flour [27]. Firstly, a reaction mixture was fabricated using 1 mL of sample (1 mg/mL), 200 µL of phenol reagent (1 N), and 1.8 mL of distilled water. The mixture was vortexed, and 3 min after, 400 µL of Na_2_CO_3_ (10 %, *v*/*v* in water) were added. Afterward, 600 µL of distilled water were added to adjust the final volume (4 mL) and left at room temperature for incubation (1 h). The absorbance was taken at 725 nm, and the phenolic content was calculated from the calibration curve of the ferulic acid standard. The content was measured as mg of ferulic acid equivalent per 100 g dry weight (dw).

### 2.5. Determination of Total Flavonoid Content

The content of total flavonoid (TF) in the processed millet was evaluated as described by Ghimeray et al. [28] with slight modifications. In brief, 500 µL of millet extract (1 mg/mL) were mixed with 100 µL of Al(NO_3_)_3_ (10%, *w*/*v*) and 100 µL of CH_3_CO_2_K (1 M) solution, and finally, 3.3 mL of DW (distilled water) were added to adjust the volume up to 4 mL. The reaction mixture was vortexed and left at room temperature for incubation (40 min). The absorbance was measured through a UV-Vis spectrophotometer at 415 nm. The total flavonoid was measured as mg/100 g of rutin equivalents on a dry weight basis.

### 2.6. Chromatographic Analysis of Phenolic Acids and Catechin 

The high-performance liquid chromatography (HPLC) condition for phenolic acids and catechin was measured following the method of Chadrasekara and Shahidi [12] and Xiang et al. [29]. Before quantifying the phenolic acid, the sample (1 mg/mL) prepared for HPLC analysis was filtered first through a 0.45 µM syringe filter (Millipore, Bedford, MA, USA). The HPLC system (CBM 20A, Shimadzu Co, Ltd., Kyoto, Japan) utilized for this study was equipped with two gradient pumps (LC 20AT, Shimadzu), one C18 column (Kinetex, 100 × 4.6 mm, 2.6 micron, Phenomenex, Torrance, CA, USA), an auto sample injector (SIL-20A, Shimadzu), a UV detector (SPD-10A, Shimadzu), and a column oven (30 °C, CTO-20A, Shimadzu). Solvent A was water containing 0.1% formic acid, and B was methanol containing 0.1% formic acid with a flow rate of 0.4 mL/min. The linear gradient was programmed, according to Chadrasekara and Shahidi [12]. The wavelengths of the absorption spectrum (lambda max) were set at 254 nm, 280 nm, and 320 nm for the phenolic acid and catechin analysis. The detected acids were calculated from the standard calibration curve of five phenolic acids standard (Daesung Chemical Machinery Ind. Co., Gyeonggi Do, South Korea) plotted by the peak area of standard samples. Stock standard solutions were prepared by dissolving the standard sample in methanol with various concentrations. The chromatographic conditions were optimized by changing the composition and proportion of the mobile phase. All samples were analyzed in triplicate, and each phenolic acid content was expressed as µg/100 g (Figure S1).

### 2.7. Antioxidant Capacity Analysis

#### 2.7.1. DPPH Free Radical Scavenging Capacity

In this analysis, DPPH (2,2-diphenyl-1 picryl hydrazyl) was used to assess the antioxidant capacity of millet extract following the method of Braca et al. [30]. The stock solution was prepared by dissolving 5.914 mg of DPPH powder in 100 mL of methanol solvent, and the absorbance range was maintained from 1.1–1.3 by a spectrophotometer. Briefly, 1 mL of millet extract was mixed with 3 mL of DPPH solution, and the mixture was left in a dark environment (at room temperature, for 30 min) after shaking vigorously. The DPPH solution (3 mL) with distilled water (1 mL) was considered the blank sample. The absorbance was measured at 517 nm through a UV-Vis spectrophotometer (UV-1800 240 V, Shimadzu Corporation, Kyoto, Japan). The scavenging capacity of the millet sample was calculated using the following formula and result expressed as a percentage:

Inhibition (%) = [(blank sample − extract sample)/blank sample] × 100

#### 2.7.2. Ferric Reducing Antioxidant Power Assay 

The reducing capacity of the millet extract was evaluated, according to Pulido et al. [31]. In this test, a reaction mixture containing 1 mL millet extract solution (1 mg/mL) and 1 mL of 0.2 M phosphate buffer (pH 6.6) was prepared, which was then left for incubation for 20 min at 50 °C. Afterward, 1 mL trichloro-acetic acid (TCA) was added slowly to the resultant mixture and centrifuged for 10 min at 3000 rpm. The supernatant was separated and added to deionized water at a 1:1 ratio followed by the addition of 250 µL of ferric chloride (0.1%) to the solution. The absorbance was measured at 700 nm by a spectrophotometer, and the result expressed as µM Fe^2+^ based on reducing the capacity of ferric chloride. 

#### 2.7.3. Phosphomolybdenum Method 

Total antioxidant capacity of millet was determined following the process of Prieto et al. [32]. In brief, 1 mL millet solution (1 mg/mL) was mixed with 3 mL of sulfuric acid (0.6 M), sodium phosphate (28 mM), and ammonium molybdate solution (4 mM), and the mixture was incubated at 95 °C for 150 min. Finally, the absorbance of the prepared mixture was taken at 695 nm, and the total antioxidant capacity was expressed as the absorbance of the sample.

#### 2.7.4. Hydroxyl Radical Scavenging Capacity

The hydroxyl radical scavenging capacity (HRSC) of the millet sample was measured according to the method descried by Halliwell et al. [33] with slight modifications. In brief, 0.1 mL of stock solution were added to a test tube containing 10 mM FeCl_3_, 10 mM EDTA, 10 mM H_2_O_2_, and 10 mM deoxyribose in potassium phosphate buffer (pH 7.4) (0.9 mL). The mixture was incubated at 37 °C for 1 h. After incubating, the sample was boiled in a water bath at 95 °C for 15 min after adding of 0.5 mL of 10% (*w*/*v*) trichloro acetic acid (TCA) and 0.5 mL of 1% (*w*/*w*) thiobarbituric acid. The absorbance was measured at 532 nm by a spectrophotometer against a blank phosphate buffer. The scavenging capacity of the millet was expressed as a percentage. 

### 2.8. Statistical Analysis

All data were expressed as the mean ± SD of triplicate measurements. The obtained results were compared among the different processing methods to observe the significant differences at the level of 5%. The paired *t*-test between the mean values of the treated samples and control were analyzed by MINITAB (Version 17.0, Minitab Inc., State College, PA, USA).

## 3. Results and Discussion

**Figure 1:**
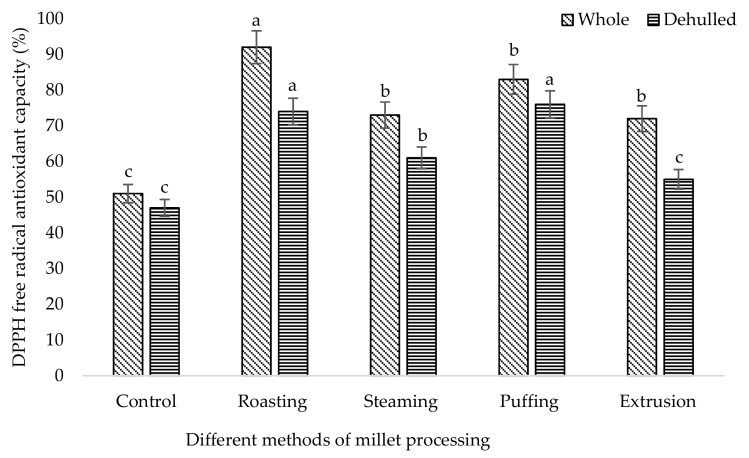
DPPH free radical antioxidant capacity of processed broomcorn millet. DPPH, 2, 2-diphenyl-1 picryl hydrazyl. The values are the mean ± SE. (*n* = 3). Different lowercase letters within the bar indicate significant differences (*p* < 0.05) according to the analysis of variance (ANOVA).

**Figure 2:**
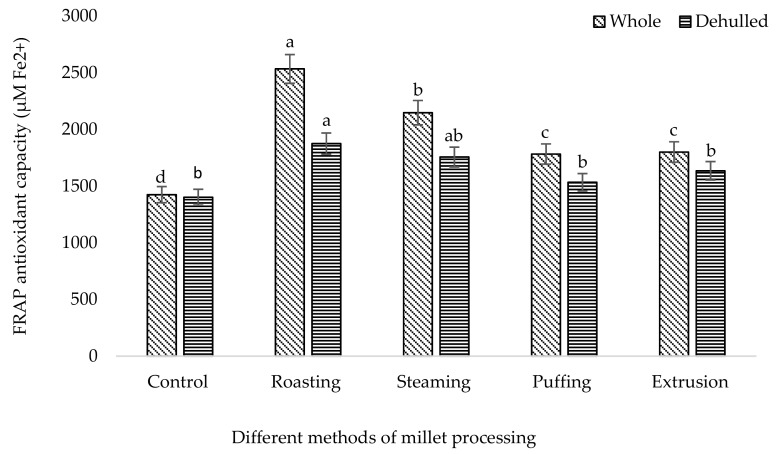
FRAP antioxidant capacity of processed broomcorn millet. FRAP: ferric reducing antioxidant power. The values are the mean ± SE. (*n* = 3). Different lowercase letters within the bar indicate significant differences (*p* < 0.05) according to ANOVA.

**Figure 3:**
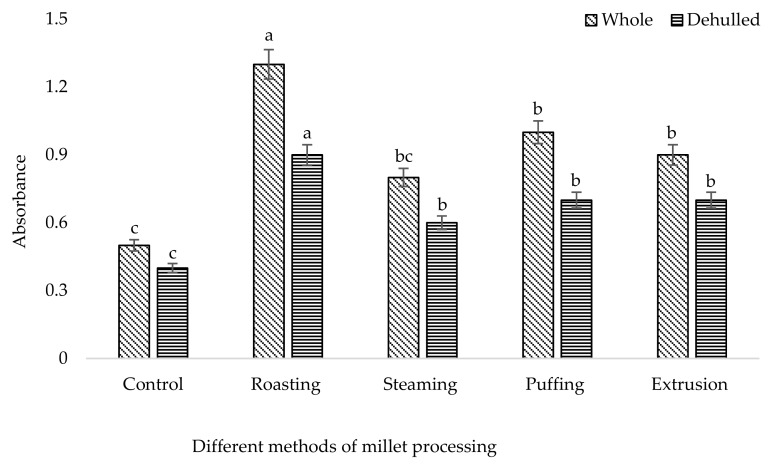
Total antioxidant capacity of processed broomcorn millet. The values are the mean ± SE. (*n* = 3). Different lowercase letters within the bar indicate significant differences (*p* < 0.05) according to ANOVA.

**Figure 4:**
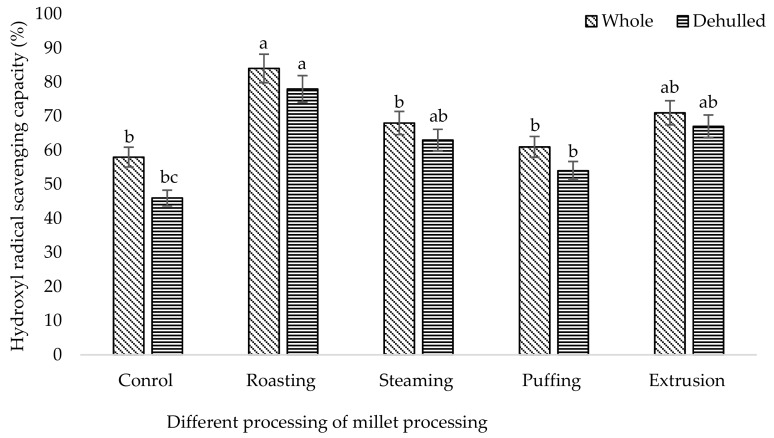
Hydroxyl radical scavenging capacity of processed broomcorn millet. The values are the mean ± SE. (*n* = 3). Different lowercase letters within the bar indicate significant differences (*p* < 0.05) according to ANOVA.

It was shown that the different processing methods had a significant effect (*p* < 0.05) on the phenolic and flavonoid content of broomcorn millet (Table 1). Among the different methods, the higher TP (670 mg/100 g) and TF (391 mg/100 g) content were accumulated in roasted whole millet followed by puffing (TP: 645 mg/100 g, TF: 304 mg/100 g), extrusion (TP: 455 mg/100 g, TF: 219 mg/100 g), and steaming (TP: 315 mg/100 g, TF:282 mg/100 g). The single phenolic acid content of broomcorn millet such as syringic acid, 4-hydroxy benzoic acid, ferulic acid, sinapic acid, and condensed tannin (catechin) was higher in roasted millet compared to the control (Table 2). The results revealed that the whole roasted millet content had higher phenolic acids including syringic acid (53.71 µg/100 g), gallic acid (62.34 µg/100 g), 4-hydroxy benzoic acid (54.19 µg/100 g), ferulic acid (118.79 µg/100 g), sinapic acid (73.25 µg/100 g), and catechin (134.24.42 µg/100 g) among the processed methods. Likewise, roasted whole millet had significantly higher antioxidant capacities compared to the control, which were analyzed by the DPPH, FRAP, PPMD, and HRSC methods (Figure 1, Figure 2, Figure 3 and Figure 4). The antioxidant capacity was measured by different methods to get consistent and accurate results.

The results exposed that different processing methods have an excellent effect on the accumulation of secondary metabolites and the antioxidant capacity of broomcorn millet. It is well established that processing enhances the accumulation of secondary metabolites in food materials [34,35]. It was shown that, among the different processing method, roasting significantly induced secondary compounds including TP, individual phenolic acids, TF, and antioxidant capacity in whole millet flour. The heat treatment of roasted millet could favor the hydrolysis of C-glycosylflavones and the release of subsequent phenolic compounds [36,37]. It was reported that the phenolic content and antioxidant capacity of the roasted finger and little millet were significantly higher compared to other processing methods [26]. Fei et al. [22] described that the content of phenolic compounds of the broomcorn millet was increased after roasting. The increased individual phenolic compound by roasting was also observed in soybean [38], buckwheat [39], Bengal gram [40], and faba beans [41]. 

The bioaccessibility of total flavonoid content from processed millet was significantly higher, and the content was about two-times superior in roasted whole millet than the control. Flavonoids were shown to modify eicosanoid biosynthesis, which caused the anti-inflammatory activity, protection of low-density lipoprotein from oxidation, and promotion of cardiovascular smooth muscle [42]. Flavonoids are also powerful antioxidants giving protection against oxidative and free radical damage. The individual flavonoids such tricin and acacetin and individual phenolic acids such as ferulic acid, caffeic acid, syringic acid, cinnamic acid, etc., have been attributed to the chemopreventive efficiency and antioxidant activities of finger millet [14,43]. Zhang et al. [11] reported that the health benefit of broomcorn millet depended on its phytochemical profiles and antioxidant properties. Kampa et al. [44] demonstrated that caffeic acid, ferulic acid, and syringic acid had antiproliferative activity against T47D human breast cancer cells. 

Pradeep et al. [26] stated that the extractability of bound phenolic compounds of millet is enhanced through thermal degradation of the cellular constitutes from insoluble ester bonds by the roasting process. The total flavonoid content was significantly increased by nearly two-times in roasted millet compared to the control. The increased flavonoid content may be due to the polymerization of the bound phenolic compound by thermal processing [45]. The increased TP and TF of millet due to roasting may have been related to the generation of Maillard reaction products (MRP) [46]. The Maillard reaction (MR) is the key series chemical reaction occurring at three different stages, early, advanced, and final stages, involving free amino groups of lysine, peptides, or proteins and the carbonyl group of reducing sugars. In the first stage of the MR reaction, amadori rearrangement products such as furosine are produced from denatured protein by heat treatment [47]. Broomcorn millet contains higher bound phenolic compounds such as ferulic acid, caffeic acid, and coumaric acid than their free counterparts; therefore, thermal processing methods promote phenolic acid to be released during treatment. Therefore, more phenolic compounds can be extracted from processed millet than control [11]. Zhan et al. [8] also reported that hydroxycinnamic acid derivatives, including ferulic acid and caffeic acid are more prevalent than the hydroxybenzoic acid derivatives, including syringic acid and gallic acid. 

Accordingly, increases in the amounts of solubilized phenolic compounds that occurred during roasting increase the antioxidant activities in plant foods. The phenolic compounds especially phenolic acids of millet are oxidized above 110 °C [48]. Considering this point, in this study, the processing temperature of the millet was fixed at 110 °C. It was also reported that total phenolic content and antioxidant capacity had a highly significant linear correlation [49]. The increases in antioxidant activity of treated samples were due to the increase of the total polyphenol and flavonoid compounds [50].

Reduction of bioactive compounds and antioxidant capacity in steaming might be due to the moisture content. It has been described that moisture content and oxygen activity in steamed millet are responsible for reducing millet bioactive compounds’ content [8]. Zhang et al. [11] also explained that bound phenolics degraded into free phenolics, and the free phenolics oxidized in the presence of high moisture content. The higher bioactive compounds and antioxidant capacity were attained in whole millet compared to dehulled millet. Vaher [50] and Kim et al. [51] reported that phenolic compounds are mainly present in cereal bran. Ivanisova et al. [52] stated that 73% of phenolic contents were found in the grain bran. The obtained results provide further support to the notion that phenolic compounds of broomcorn millet are concentrated in the bran.

Data of the antioxidant properties of the processed millet determined by DPPH, FRAP, total antioxidant capacity, and HRSC confirmed that all of the processed millet had higher peroxide scavenging capacity than the control. The result showed that the processing method affects the antioxidant properties of millet. Antioxidant capacity is increased using stopping free radical chain reactions by the hydrogen donating properties of the phenolic hydroxyl groups [53]. DPPH is a stable free radical used to determine the antioxidant properties or radical scavenging capacity. Our findings indicated that roasted millet represents a significant source of antioxidants, which supports their potential as a natural functional food. A similar result of antioxidant capacity was also ascribed for barley [54], beans [55], buckwheat [39], and soybean [36] upon roasting treatment. 

The higher antioxidant capacity of the roasted millet is due to the breakdown of cellular constituents and membranes. The Maillard reaction occurs during heating and allows the formation of different intermediate byproducts, which might contribute to the antioxidant properties [50,56]. Moreover, previously, it was reported that dark color pigments, particularly melanoidins, are produced during the thermal processing of foods, which are extensively known to have the antioxidant capacity [46]. From the current study, a strong correlation between phenolic compounds and antioxidant properties in millet was shown, which was also supported by the previous study of *Angelica gigas* Nakai [49]. However, Gujral et al. [40] and Sharma and Gujral [54] observed a poor positive correlation between total phenolic compounds and antioxidant capacity in roasted Bengal gram and barely, respectively.

## 4. Conclusions

The health benefits of millets are already well recognized. The present study clearly showed that roasting significantly increased the content of secondary compounds and the antioxidant properties of the broomcorn millet. It is also showed that the whole millet grain possessed more phenolic compounds and antioxidant capacity than the dehulled millet. In all treatments, HPLC manifested a higher amount of ferulic acid among other acids. Finally, it might be concluded that processing treatments have a significant effect; especially roasting can be considered a suitable processing method to enhance functionality and improve the nutraceutical compounds of whole broomcorn millet.

## Figures and Tables

**Table 1:** Total phenolic and flavonoid content of processed broomcorn millet

Treatments	Total Phenolic Content (mg/100 g Ferulic Acid Equi.) dw		Total Flavonoid Content (mg/100 g Rutin Equi.) dw	
	Whole	Dehulled	Whole	Dehulled
Control	295 ± 2.34 ^d^	167 ± 1.23 ^e^	183 ± 3.57 ^c^	154 ± 4.53 ^d^
Roasting	670 ± 1.57 ^a^	587 ± 1.87 ^a^	391 ± 3.26 ^a^	301 ± 5.31 ^a^
Steaming	315 ± 3.48 ^d^	274 ± 2.14 ^d^	282 ± 2.58 ^b^	212 ± 5.21 ^c^
Puffing	645 ± 2.35 ^b^	547 ± 2.56 ^a^	304 ± 4.25 ^b^	256 ± 3.25 ^b^
Extrusion	455 ± 1.64 ^c^	315 ± 1.26 ^c^	219 ± 3.68 ^bc^	167 ± 5.14 ^d^

Each value is expressed as the mean ± SD (*n* = 3). Values labeled with different letters in a column are significantly different (*p* < 0.05). dw: dry weight basis.

**Table 2:** Analysis of phenolic acids and catechin of processed millet by different methods.

Treatments	Hydroxybenzoic Acid Derivatives						Hydroxycinnamic Acid Derivatives				Flavonoid (Condensed Tannin)	
	Syringic Acid (μg/100 g)		Gallic Acid (µg/100 g)		4 Hydroxy Benzoic Acid (µg/100 g)		Ferulic Acid (µg/100 g)		Sinapic Acid (µg/100 g)		Catechin (µg/100 g)	
	Whole	Dehulled	Whole	Dehulled	Whole	Dehulled	Whole	Dehulled	Whole	Dehulled	Whole	Dehulled
Control	11.27 ± 2.7 ^c^	5.34 ± 0.8 ^d^	9.37 ± 4.1 ^c^	11.1 ± 0.8 ^c^	9.11 ± 3.4 ^c^	6.87 ± 0.4 ^c^	68.63± 9.4 ^c^	38.36 ± 1.8 ^d^	9.32 ± 1.9 ^d^	12.87 ± 3.5 ^d^	88.48 ± 12.3 ^b^	90.7 ± 11.4 ^a^
Roasting	53.71 ± 2.2 ^a^	32.45 ± 5.9 ^b^	62.34 ± 3.4 ^a^	25.72 ± 4.3 ^b^	54.19 ± 5.1 ^a^	24.11 ± 4.6 ^a^	118.79 ± 13.7 ^a^	94 ± 5.4 ^a^	73.25 ± 11.8 ^a^	56.2 ± 8.2 ^a^	134.24 ± 11.3 ^a^	94 ± 7.9 a
Steaming	49.03 ± 6.5 ^a^	56.37 ± 6.6 ^a^	39.86 ± 7.7 ^b^	39 ± 7.1 ^a^	8.1 ± 3.5 ^c^	6.9 ± 0.5 ^b^	57.37 ± 6.3 ^c^	47.75 ± 4.6 ^c^	43 ± 9.2 ^b^	29 ± 7.4 ^b^	52.94 ± 13.7 ^c^	88 ± 13.7 ^b^
Puffing	31.42 ± 3.19 ^b^	34.6 ± 5.7 ^b^	28.69 ± 5.8 ^b^	23.06 ± 6.4 ^b^	49.76 ± 3.7 ^a^	27.16 ± 7.3 ^a^	88.6 ± 13.7 ^b^	93.47 ± 9.2 ^a^	27 ± 6.3 ^c^	19 ± 3.7 ^c^	104.4 ± 14.8 ^b^	73.3 ± 15.3 ^b^
Extrusion	28.21 ± 2.4 ^b^	19.01 ± 7.3 ^c^	39.29 ± 3.4 ^b^	23.62 ± 6.2 ^b^	23.63 ± 6.8 ^b^	6.52 ± 7.9 ^b^	79.22 ± 8.3 ^b^	63 ± 9.3 ^b^	22 ± 3.8 ^c^	17 ± 2.8 ^c^	84.78 ± 9.7 ^b^	98.48 ± 11.7 ^a^

Each value is expressed as the mean ± SD (*n* = 3). Values labeled with different letters in a column are significantly different (*p* < 0.05).

## Supplementary Materials

The following are available online at https://www.mdpi.com/2304-8158/8/7/230/s1, Figure S1: HPLC chromatograms.
