# Supplementary material for: Effect of Different Processing Methods on the Accumulation of the Phenolic Compounds and Antioxidant Profile of Broomcorn Millet (Panicum miliaceum L.) Flour
Source: Foods. 2019 Jun 27;8(7):230. doi: 10.3390/foods8070230 (PMC6678386; doi:10.3390/foods8070230)
Supplement: Supplementary file 1 [file foods-08-00230-s001.pdf]

Article

Effect of Different Processing Methods on the Accumulation of the Phenolic compounds and Antioxidant Profile of Broomcorn Millet (*Panicum miliaceum* L.) Flour

Md Obyedul Kalam Azad [1,2], Da In Jeong [1], Md Adnan [1], Timnoy Salitxay [3], Jeong Won Heo [1], Most Tahera Naznin [4], Jung Dae Lim [5], Dong Ha Cho [1], Byoung Jae Park [6] and Cheol Ho Park [1,*]

1. Department of Bio-Health Technology, College of Biomedical Science, Kangwon National University, Chuncheon, 24341, Korea; azadokalam@gmail.com (M.O.K.A.); jdi5463@hanmail.net (D.I.J.); hjw9689@naver.com (J.W.H.); mdadnan1991.pharma@gmail.com (M.A.); chodh@kangwon.ac.kr (D.H.C.)
2. Head of Research and Technology, Rentia Plant Factory, Chuncheon, 24341, Korea
3. Laos- Korea Science and Technology Center, Souphanouvong University, Luangprabang, 0603, Laos; timnoy555@gmail.com
4. Department of Biosystems and Technology, Swedish University of Agricultural Sciences, Box 103 23053 Alnarp, Sweden; naznin.most.tahera@slu.se
5. Department of Herbal Medicine Resource, Kangwon National University, Samcheok 25949, Korea; ijdae@kangwon.ac.kr
6. Faculty of Agriculture, Kagoshima University, Kagoshima, 890-0065, Japan; seabass80@hanmail.net

* Correspondence: chpark@kangwon.ac.kr; Tel.: +82-33-259-5642

Supplemental file:

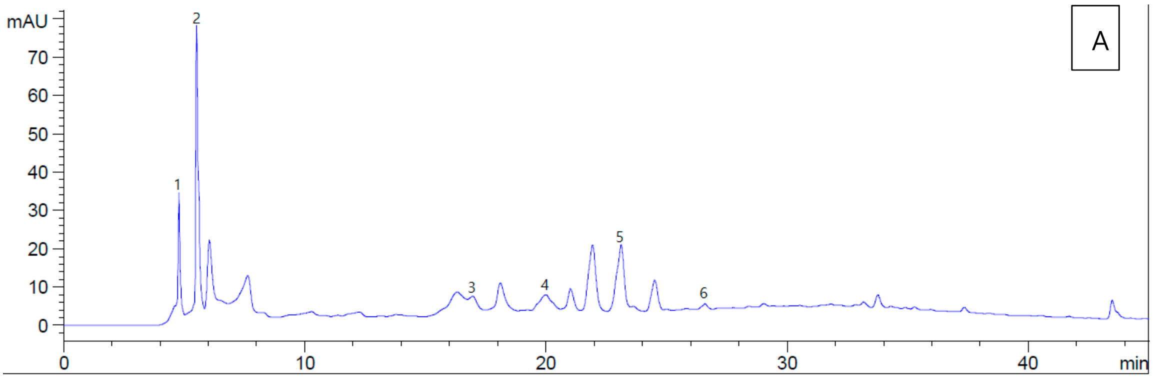

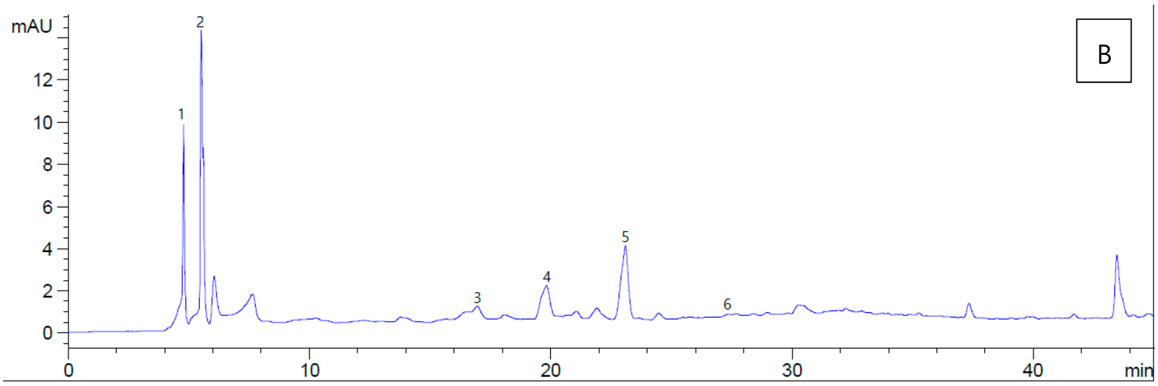
mAU
12
10
8
6
4
2
0
1
2
3
4
5
6
0
10
20
30
40
min
B

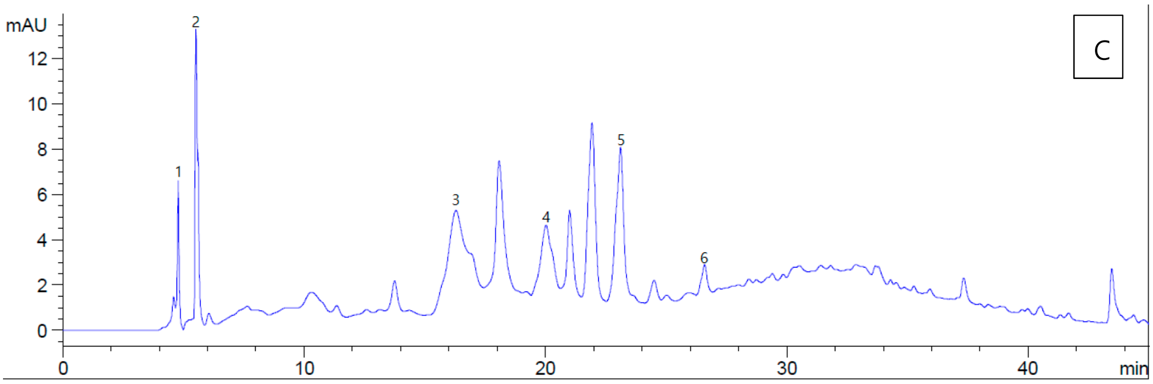
mAU
12
10
8
6
4
2
0
1
2
3
4
5
6
0
10
20
30
40
min
C

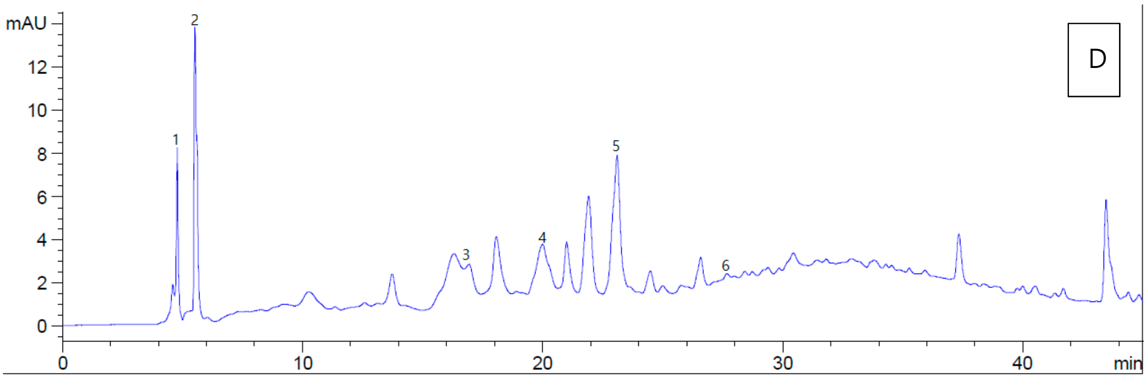
mAU
12
10
8
6
4
2
0
1
2
3
4
5
6
0
10
20
30
40
min
D

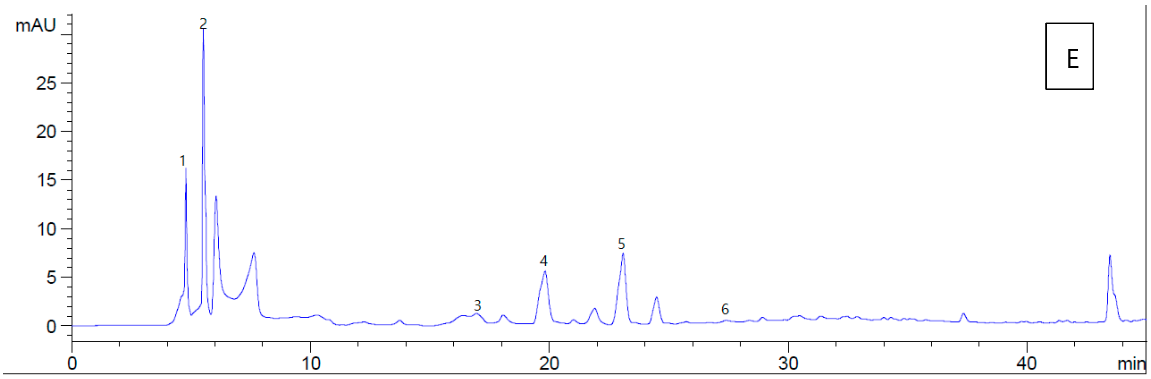
mAU
25
20
15
10
5
0
1
2
3
4
5
6
0
10
20
30
40
min
E

F

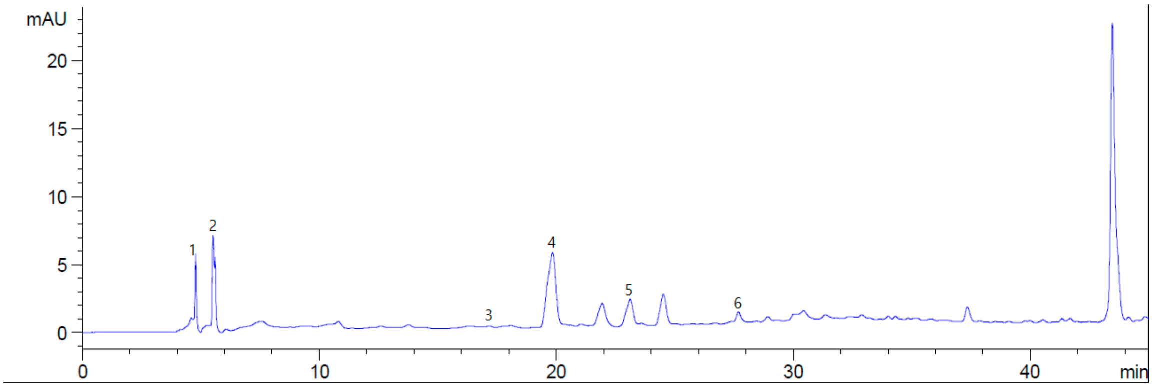
mAU
20
15
10
5
0
1
2
3
4
5
6
0
10
20
30
40
min

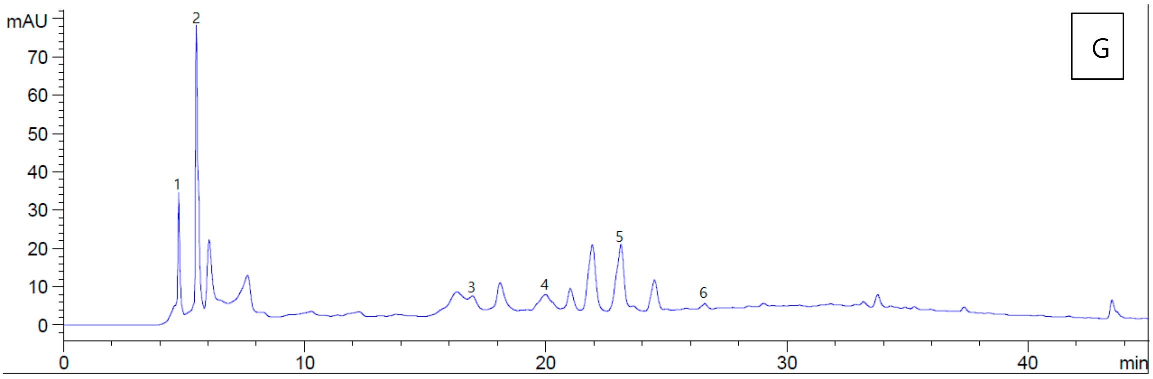
G
mAU
70
60
50
40
30
20
10
0
1
2
3
4
5
6
0
10
20
30
40
min

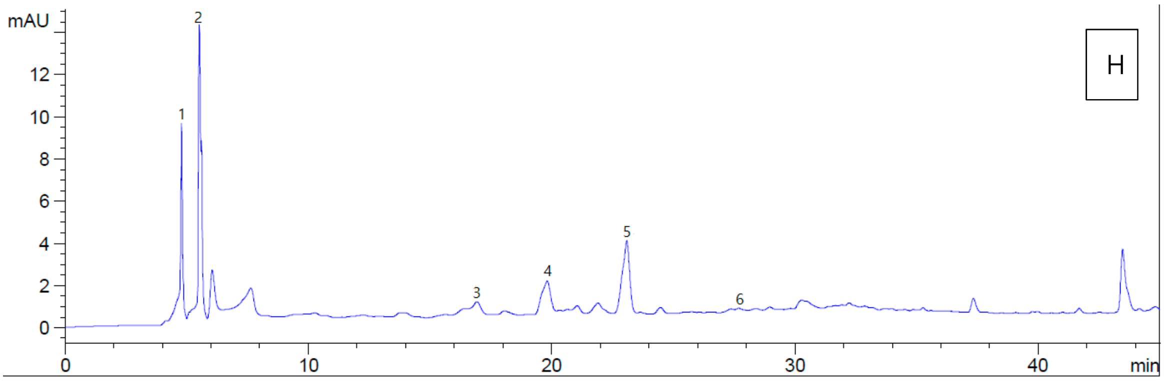
H
mAU
12
10
8
6
4
2
0
1
2
3
4
5
6
0
10
20
30
40
min

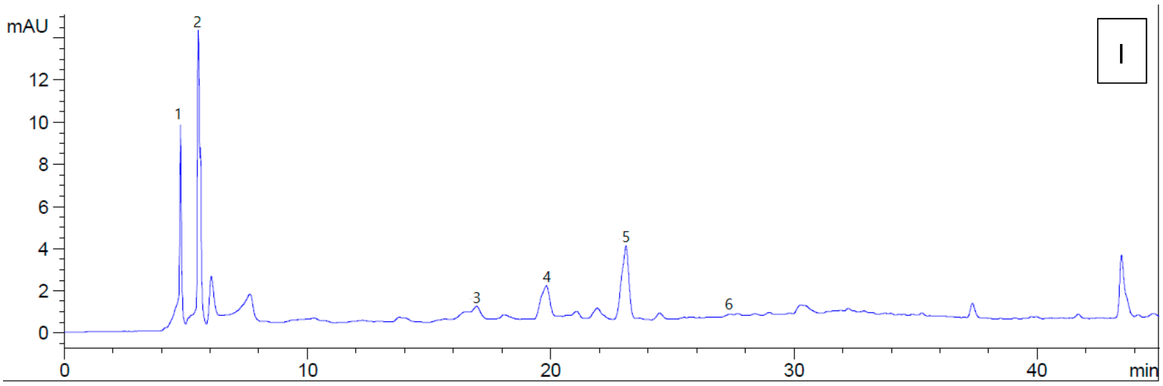
I
mAU
12
10
8
6
4
2
0
1
2
3
4
5
6
0
10
20
30
40
min

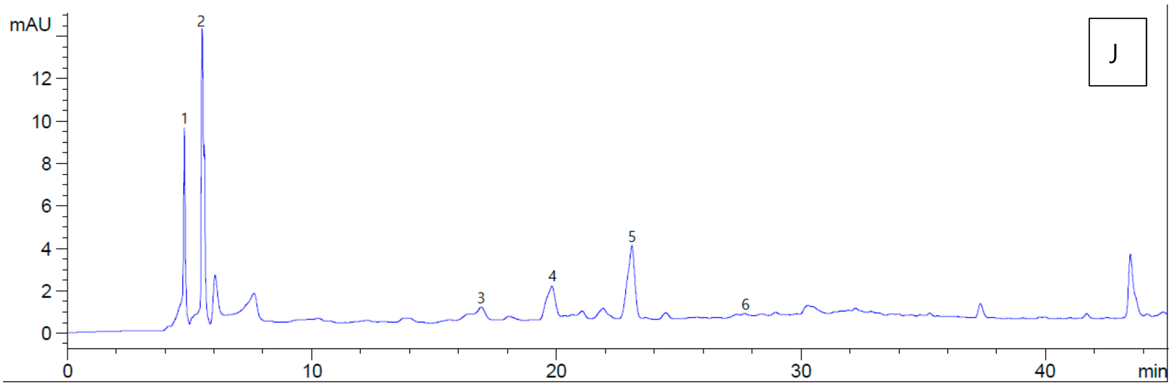

Figure S 1. HPLC chromatograms. 1. Gallic acid 2. Catechin 3. 4-hydroxy benzoic acid 4. Syringic acid 5.Ferulic acid 6.Sinapic acid

A. Control whole B. Control dehulled C. Roasting whole D. Roasting dehulled E. Steaming whole F. Steaming dehulled G.Puffing whole H. Puffing dehulled I. Extrusion whole J. Extrusion dehulled

Note: The wavelengths of the absorption spectrum (lambda max) were set at 254 nm, 280 nm, and 320 nm for the phenolic acid and catechin analysis. However, most of the peaks were found well separated and sharp at 320 nm wavelength. Therefore, the chromatograms produced at 320 nm were considered for quantitative calculations.
